# Case of Cartilaginous Structure of the Cervix Uteri in a Pregnant Woman, Requiring an Incision through the Os Uteri to Render Delivery Practicable

**Published:** 1829

**Authors:** G. Cotton

**Affiliations:** Marietta, President of the Medical Society of Ohio


**Art. II.**—*Case of Cartilaginous Structure of the Cervix Uteri in a pregnant woman, requiring an incision through the Os Uteri to render delivery practicable.* By G. Cotton, M. D. of Marietta, President of the Medical Society of Ohio.

I was called to visit Mrs. S—— about twelve miles from Marietta, while in labour with her second child in consultation with Dr. B——. She had then suffered more than forty eight hours with severe pains. On examination, I found the os Uteri about two inches within the os Externum, and dilated to the size of half a dollar; but instead of the soft and yielding appearance usual at such times, I was surprised to find it hard and rigid, and apparently of a cartilaginous structure. The cervix Uteri did not as usual project into the Vagina, but was continous with it. I then understood that she had suffered very greatly in her former labour, and was at length delivered with instruments. Much inflammation followed, which resulted in a sloughing of a portion of the internal parts of the Vagina, and probably a portion of the cervix Uteri; after which, adhesions took place in such manner as to obliterate the projecting neck of the Uterus. Dr. B. informed me that the os Uteri had remained in the present state for at least forty eight hours.

We concluded to try the effect of copious blood letting, which is oftentimes so efficacious in producing a rel[illegible]ation of the os Uteri, though I expected but little benef[illegible] from the operation.

We accordingly took from a large orifice in the arm, about thirty two ounces of blood, which produced considerable faintness, but not the slightest alteration in the state of the os Uteri.

In the mean time, the woman had become much exhausted, indeed she had been, for a long time in feeble heath, and had been supposed to be dropsical.

No hope now remained of effecting a delivery by ordinary means. Under these circumstances we concluded to make an incision through the rigid os Uteri, sufficient to enable us to deliver with the crotchet. Accordingly I made a longitudinal incision of about one and a half or two inches in length through the cartilaginous structure of the cervix Uteri, selecting that part which seemed most rigid; and by using the Crotchet was enabled to bring down the child which was small and in a half putrid state. No unusual hæmorrhage occurred. The placenta however we could not deliver; and after several ineffectual attempts, the cord having separated from it, and the woman becoming much exhausted, with laborious respiration, and other unfavourable symptoms, we concluded to leave her to repose; thinking that the placenta would probably be ejected by the action of the Uterus, when it should recover from its present exhaustion. The next morning she was much recovered, and in the course of the day the placenta was expelled. This woman afterward recovered without the occurrence of any very unfavourable symptoms.

This case exhibits the danger of an incautious use of instruments in the practice of midwifery, as there can be no doubt that the sloughing and consequent adhesions which occurred subsequent to her former confinement, had arisen from that cause.

